# Mental Health Care Utilization Among Parents of Children With Cancer

**DOI:** 10.1001/jamanetworkopen.2024.4531

**Published:** 2024-04-02

**Authors:** Xin Hu, Scott D. Grosse, Xuesong Han, Jordan Gilleland Marchak, Xu Ji

**Affiliations:** 1 Department of Public Health Sciences, University of Virginia School of Medicine, Charlottesville; 2 National Center on Birth Defects and Developmental Disabilities, Centers for Disease Control and Prevention, Atlanta, Georgia; 3 Surveillance and Health Equity Science, American Cancer Society, Atlanta, Georgia; 4 Department of Pediatrics, Emory University School of Medicine, Atlanta, Georgia; 5 Aflac Cancer and Blood Disorders Center, Children’s Healthcare of Atlanta, Atlanta, Georgia

## Abstract

**Importance:**

Caring for children diagnosed with cancer may adversely affect the mental health (MH) of parents.

**Objective:**

To characterize utilization of MH services among parents of children with vs without cancer using nationwide commercial claims data.

**Design, Setting, and Participants:**

For this cross-sectional study, the Merative MarketScan Commercial Claims Database was used to identify continuously insured families of children treated for cancer (aged ≤21 years at diagnosis) during 2010 to 2018, compared with families who matched eligibility criteria but did not have a child with a cancer history. Parents were assessed from 18 months before to 12 months after their child’s cancer diagnosis. Analyses were conducted from February 2022 to September 2023.

**Exposures:**

Children’s cancer diagnosis.

**Main Outcomes and Measures:**

Outcomes included parents’ MH-related visits during the first year following their child’s cancer diagnosis. Logistic regressions compared outcomes between families of children with vs without cancer, adjusting for sociodemographic and clinical factors.

**Results:**

This study included 4837 families of children with cancer (4210 mothers and 4016 fathers) and 24 185 families of children without cancer (21 444 mothers and 19 591 fathers) with continuous insurance enrollment. Most household leads were aged 35 to 54 years (3700 [76.5%] in families of children with cancer vs 17 812 [73.6%] in families of children without cancer) and resided in urban areas (4252 [87.9%] vs 21 156 [87.5%]). The probabilities of parents having anxiety-related visits (10.6% vs 7.0%), depression-related visits (8.4% vs 6.1%), and any MH-related visits (18.1% vs 13.3%) were higher in families of children with vs without cancer. Adjusted analyses showed absolute increases of 3.2 percentage points (95% CI, 2.3 to 4.0; 45.7% relative increase), 2.2 percentage points (95% CI, 1.4 to 3.0; 36.1% relative increase), and 4.2 percentage points (95% CI, 3.1 to 5.3; 31.3% relative increase) in the probabilities of 1 or both parents having anxiety-related visits, depression-related visits, and any MH-related visits, respectively, among families of children with vs without cancer. Such differences were greater in magnitude among mothers than fathers.

**Conclusions and Relevance:**

In this cohort study of privately insured parents, those caring for children with cancer had a higher likelihood of utilizing MH care than other parents. These findings underline the importance of interventions toward targeted counseling and support to better meet MH care needs among parents and caregivers of children with cancer.

## Key Points

**Question:**

Are parents caring for children diagnosed with cancer more likely to utilize mental health (MH) services than other parents?

**Findings:**

In this cross-sectional study of 4837 privately insured families caring for children diagnosed with cancer during 2010 to 2018, there were statistically significant increases in the probabilities of 1 or both parents having anxiety-related, depression-related, and any MH-related visits, respectively, compared with families of children without cancer. Such differences were greater in magnitude among mothers than fathers.

**Meaning:**

These findings suggest that targeted interventions to provide counseling and support are warranted to better meet MH care needs among parents and caregivers of children with cancer.

## Teaser

This cross-sectional study uses US commercial health insurance claims data to assess mental health care utilization among parents of children with vs without cancer.

## Introduction

In 2021, approximately 15 590 children (aged 0-19 years) in the US were diagnosed with cancer.^1^ Despite medical advances that have improved the 5-year survival rate of pediatric cancer,^2,3^ cancer remains the third leading cause of death among children.^4^ The morbidity and mortality associated with pediatric cancer and difficulties navigating cancer care may negatively affect the mental health (MH) of parents and caregivers.^5,6^ The prevalence of clinically relevant anxiety and depression among parents of children with cancer is as high as 74% and 46%, respectively.^7,8,9^

Pediatric cancer treatment is complex. Parents provide informal care to their child during and after cancer therapy,^10^ and they must balance competing priorities such as work and caregiving to other family members.^11^ In addition to witnessing their child’s suffering, the uncertainty of cancer prognosis and financial hardship resulting from medical expenses and lost income may intensify parental distress and trigger MH problems^11,12,13^ and may also become barriers to parents seeking needed MH care. Without timely effective treatment, MH conditions may not only drive morbidity and mortality among parents but also undermine the well-being of their children.^14,15^

Professional societies have developed standards of care that recommend routine psychosocial assessment and interventions for caregivers of children with cancer.^16^ Although studies have examined self-reported MH status in caregivers of children with cancer,^10,12,13,16,17,18^ empirical evidence of recommended psychosocial service utilization among caregivers is lacking. We hypothesized that utilization of MH services would be higher among parents of children with cancer than in the general parent population.

## Methods

### Data and Sample

For this cross-sectional study, we used 2009 to 2019 data from the Merative MarketScan Commercial Claims Database, a nationwide convenience sample of inpatient, outpatient, and pharmacy insurance claims data from employer-sponsored health plans in the US.^19^ This study was approved by the Emory University Institutional Review Board under expedited review, and informed consent was waived because deidentified claims data were used. The study followed the Strengthening the Reporting of Observational Studies in Epidemiology (STROBE) reporting guideline.

We derived a sample of children (N = 15 352) diagnosed with common pediatric cancers (hematologic, bone and soft tissue, central nervous system [CNS], or gonadal) at age 21 years or younger^20^ between July 1, 2010, and December 31, 2018. These cancer types were identified by algorithms applied in previous claims databased studies,^21,22,23,24^ representing the majority of incident pediatric cancer cases in the US.^25^ To define cancer diagnosis, we required 2 or more outpatient or inpatient health care claims on distinct dates with relevant *International Classification of Diseases, Ninth Edition* or *Tenth Edition*, *Clinical Modification* codes that fell within corresponding Clinical Classification Software categories (eTable 1 in Supplement 1).^26,27^ The date of the first claim with a cancer diagnosis was the index date. We further restricted the search to children with claims associated with cancer therapies (surgery, chemotherapy, radiotherapy, or hematopoietic stem cell transplantation [HSCT]) using procedure codes, National Drug Codes, or both.^21,22,23^

For each child with cancer, we identified their parents in the same insurance plan using the family identification number and information indicating the parents’ relationship with the child. In a sensitivity analysis of families with both parents identified, results were similar. Of the 14 719 families with 1 or both parents identified, we excluded 8098 families without continuous insurance enrollment from 18 months before through 12 months after the index date, 41 families with parents aged younger than 25 or older than 64 years (because we did not have Medicare data), 189 families with more than 2 parents and 9 families with same-sex parents (due to complicated family structure and small numbers), 214 families with missing information on geographic region or rurality, and 1331 families with plans that carved out MH services (eFigure 1 in Supplement 1).^28^ The 9882 excluded families had higher proportions who were enrolled in preferred provider organization (PPO) plans and were caring for younger children, children with hematologic cancers, and children receiving chemotherapy than families in our analytic sample; other sample characteristics were similar (eTable 2 in Supplement 1).

Additionally, we identified a sample of families that did not have a child with cancer. These families were matched to families of children with cancer, at a 5:1 ratio, based on child birth year, sex, and geographic region.^28,29^ Each family of children without cancer was assigned the index date of their matched family. We applied the same inclusion and exclusion criteria to families of children without cancer (eMethods in Supplement 1).

### Outcomes

For each family, dichotomous indicators were created to assess any parent with visits related to an MH condition during the year after the index date, including anxiety, depression, substance use and related disorder (SUD), and any MH condition (ie, any of the aforementioned conditions). An MH condition was defined as a parent having 1 or more inpatient claims, 2 or more outpatient claims, or both, on distinct dates, with the corresponding diagnosis codes (eTable 3 in Supplement 1) during the year following the index date.^30,31,32,33^ For parent-level analyses, MH outcomes assessed whether the father (or mother) had visits related to an MH condition separately. In a supplemental analysis, we classified parents as having no visit, an initial visit only (ie, only 1 inpatient visit or only 2 outpatient visits), or visits in addition to the initial visit (ie, additional visits) for an MH condition during the year following the index date.

### Covariates

Sociodemographic characteristics included family geographic region, rurality of residence, parent age, child sex, child age at index date, number of children in the family, and health plan type. Health plan type was classified as high-deductible plan, health maintenance organization (HMO), PPO, and other plan types.^34^ Other covariates included parent MH history and Charlson Comorbidity Index score.^35^ Parent MH history was defined as having an MH condition from 18 months to 6 months before the index date. Subgroup analyses among families of children with cancer also included child cancer type and treatment modalities.

### Statistical Analysis

We compared study outcomes and covariates between families of children with vs without cancer using the *t* test and the χ^2^ test. Multiple logistic regressions were conducted to estimate the adjusted probability difference of having anxiety-related, depression-related, SUD-related, or any MH-related visits between families of children with vs without cancer, adjusting for study covariates and index year. We further conducted regressions for fathers and mothers separately. In the supplemental analysis, we estimated generalized ordered logistic regression to compare the probability of additional visits (vs no or only initial visit) between families of children with vs without cancer. Among parents of children with cancer, we conducted multiple logistic regressions to identify factors associated with parent MH-related visits, including the aforementioned covariates and child cancer type and treatment modalities.

Marginal effects (MEs) were generated using the margins command in Stata, version 16.0 (StataCorp). Marginal effects were interpreted as the difference in model-adjusted percentages of families or parents caring for children with cancer who had MH-related visits compared with families or parents of children without cancer.^7^ For ease of interpretation, we also calculated relative increases for each outcome, dividing the ME by the adjusted probability among families or parents of children without cancer. To compare sample characteristics, *P* < .05 was used as the threshold to determine statistical significance, with 2-sided tests. Results that accounted for multiple testing for 4 outcomes using the very conservative Bonferroni adjustment^36^ at *P* < .0125 were consistent with the findings using *P* < .05. Analyses were conducted from February 2022 to September 2023.

## Results

### Sample Characteristics

Our final analytic sample included 4837 families of children with cancer (4210 mothers and 4016 fathers) and 24 185 families of children without cancer (21 444 mothers and 19 591 fathers). Most families of children with vs without cancer had both mothers and fathers identified under the same health plans (3389 [70.1%] vs 16 850 [69.7%]; Table 1). A higher proportion of families caring for children with cancer had 3 or more children in the household compared with families of children without cancer (1998 [41.3%] vs 6092 [25.2%]). Other characteristics were similar between the 2 groups; most families of children with vs without cancer resided in urban areas (4252 [87.9%] vs 21 156 [87.5%]), most household leads were aged 35 to 54 years (3700 [76.5%] vs 17 812 [73.6%]), more than half of children were male (2837 [58.7%] vs 14 185 [58.7%]), and nearly half of children were aged 15 to 21 years at the index date (2307 [47.7%] vs 11 535 [47.7%]).

**Table 1.** Characteristics of Families Caring for Children With vs Without Cancer^a^

Characteristic	With cancer (n = 4837)	Without cancer (n = 24 185)	*P* value^b^
**Parent**			
Insurance enrollment status			
Mother only	821 (17.0)	4594 (19.0)	<.001
Father only	627 (13.0)	2741 (11.3)	
Both mother and father	3389 (70.1)	16 850 (69.7)	
Age group, y^c^			
25-34	543 (11.2)	2975 (12.3)	.001
35-44	1781 (36.8)	8514 (35.2)	
45-54	1919 (39.7)	9298 (38.4)	
55-64	594 (12.3)	3398 (14.1)	
Rurality of residence			
Rural	585 (12.1)	3029 (12.5)	.41
Urban	4252 (87.9)	21 156 (87.5)	
US geographic region^d^			
Northeast	1109 (22.9)	5545 (22.9)	>.99
Midwest or North Central	1156 (23.9)	5780 (23.9)	
South	1799 (37.2)	8995 (37.2)	
West	773 (16.0)	3865 (16.0)	
Health plan type			
High deductible	616 (12.7)	3053 (12.6)	.005
HMO	526 (10.9)	2988 (12.4)	
PPO	2473 (51.1)	11 818 (48.9)	
Other^e^	1222 (25.3)	6326 (26.2)	
No. of children in household			
1	702 (14.5)	7333 (30.3)	<.001
2	2137 (44.2)	10 760 (44.5)	
≥3	1998 (41.3)	6092 (25.2)	
CCI score, mean (SD)^c^	0.2 (0.6)	0.2 (0.7)	.007
Presence of mental health history^c^^,^^f^	639 (13.2)	2866 (11.9)	.008
**Child**			
Age at index date, y^g^			
0-4	860 (17.8)	4300 (17.8)	>.99
5-14	1670 (34.5)	8350 (34.5)	
15-21	2307 (47.7)	11 535 (47.7)	
Sex			
Male	2837 (58.7)	14 185 (58.7)	>.99
Female	2000 (41.3)	10 000 (41.3)	
Year of index date^g^			
2010	362 (7.5)	1810 (7.5)	>.99
2011	796 (16.5)	3980 (16.5)	
2012	676 (14.0)	3380 (14.0)	
2013	646 (13.4)	3230 (13.4)	
2014	521 (10.8)	2605 (10.8)	
2015	577 (11.9)	2885 (11.9)	
2016	514 (10.6)	2570 (10.6)	
2017	389 (8.0)	1945 (8.0)	
2018	356 (7.4)	1780 (7.4)	
Cancer type			
Hematologic	2350 (48.6)	NA	NA
Bone and soft tissue	627 (13.0)	NA	NA
CNS	1119 (23.1)	NA	NA
Gonadal	500 (10.3)	NA	NA
Multiple types	241 (5.0)	NA	NA
Cancer treatment modality^h^			
Any HSCT	271 (5.6)	NA	NA
Any radiation (no HSCT)	1334 (27.6)	NA	NA
Any chemotherapy (no HSCT or radiation)	2199 (45.5)	NA	NA
Surgery only	1033 (21.4)	NA	NA

Abbreviations: CCI, Charlson Comorbidity Index; CNS, central nervous system; HMO, health maintenance organization; HSCT, hematopoietic stem cell transplantation; NA, not applicable; PPO, preferred provider organization.

^a^ Data are presented as No. (%) of families. For this analysis of the Merative MarketScan Commercial Claims Database, families were identified by the family identification number from the insurance plan, and parents within the families were identified using information indicating the parents’ relationship with the child. Percentages have been rounded and therefore may not total 100.

^b^ A χ^2^ test or Fisher exact test was used to compare percentages, and the *t* test was used to compare means between families of children with vs without cancer.

^c^ For family-level analysis, these characteristics (ie, parent age group, parent CCI score, and parent mental health history) refer to the characteristics of the household lead.

^d^ Follows the categorization defined by the US Census Bureau.^37^

^e^ Includes basic or major medical plan, comprehensive plan, exclusive provider organization, noncapitated point-of-service plan, and capitated or partially capitated point-of-service plan.

^f^ Defined as having any mental health condition during the recall period from 18 months to 6 months before the index date.

^g^ Index date for a child with cancer was defined as the date of the first health care claim with a cancer diagnosis. Index date for a child without cancer was assigned as the index date of their matched child with cancer.

^h^ To measure children’s treatment modality, a mutually exclusive categorical variable was created that follows a hierarchical coding strategy to classify children with cancer into the following 4 groups who (1) received any HSCT; (2) did not receive any HSCT but received any radiation therapy; (3) did not receive any HSCT or radiation but received any chemotherapy; and (4) did not receive any HSCT, radiation, or chemotherapy but received surgery only.

Among families of children with cancer, the most common diagnosis was hematologic cancer (2350 [48.6%]), followed by CNS cancer (1119 [23.1%]) and bone and soft tissue cancer (627 [13.0%]; Table 1). There were 271 HSCT recipients (5.6%); among those who did not receive HSCT, 1334 (27.6%) received radiation therapy, 2199 (45.5%) received chemotherapy but not radiation therapy, and 1033 (21.4%) received surgery only.

### MH-Related Visits Among Families of Children With vs Without Cancer

**Figure 1.**
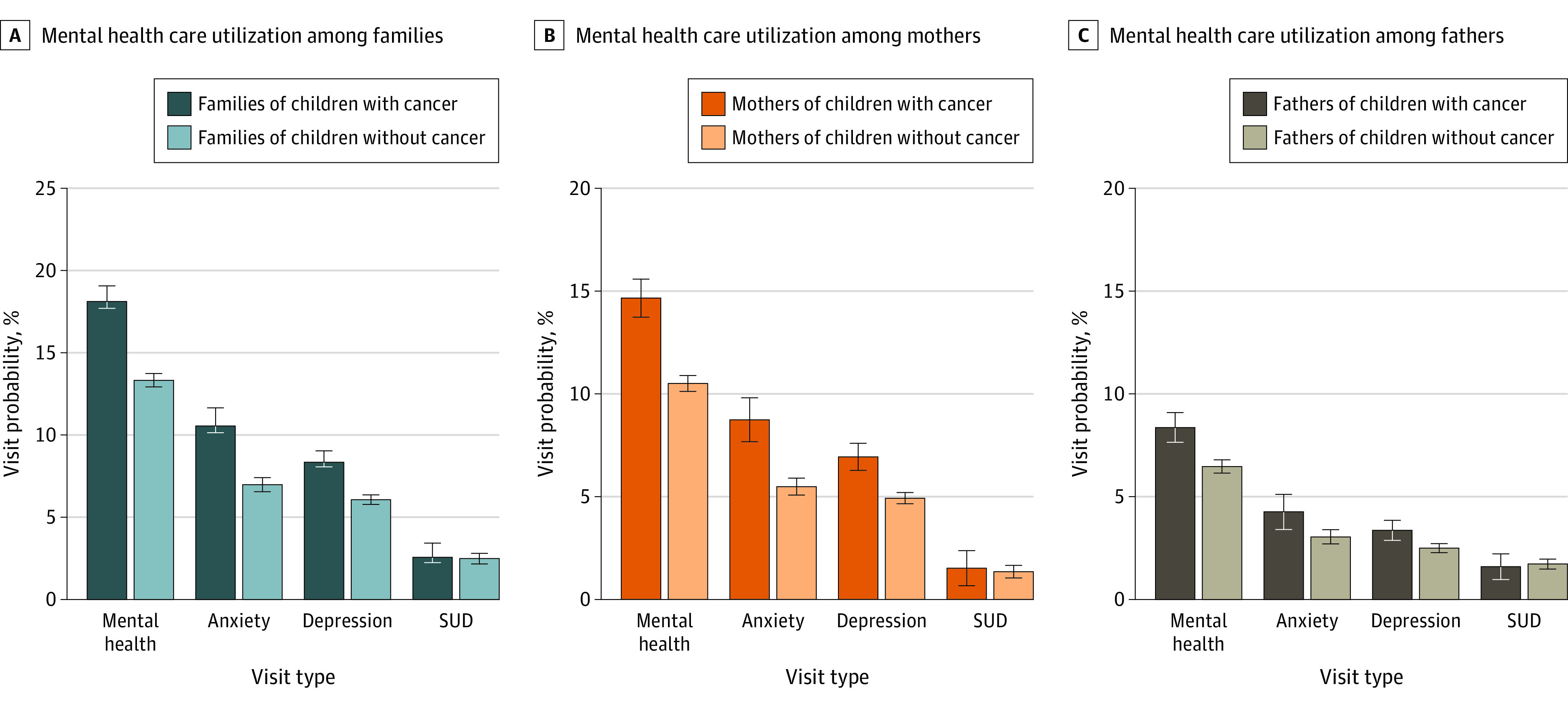
Unadjusted Probability of Mental Health–Related Visits During the Year Post Index Date Among Families or Parents Caring for Children With vs Without Cancer Analysis of the Merative MarketScan Commercial Claims Database for families (A), mothers (B), and fathers (C). SUD indicates substance use or related disorder. Error bars indicate 95% CIs.

Unadjusted analysis showed higher proportions of MH-related visits among families of children with vs without cancer, including anxiety-related visits (551 [10.6%] vs 1688 [7.0%]), depression-related visits (404 [8.4%] vs 1468 [6.1%]), and any MH-related visits (876 [18.1%] vs 3224 [13.3%]) during the year post index (Figure 1A). Proportions of SUD-related visits were similar between families of children with vs without cancer (124 [2.6%] vs 601 [2.5%]). Supplemental analysis showed that for any MH-related visits among families of children with vs without cancer, 240 (5.0%) vs 929 (3.8%) had an initial visit only and 636 (13.1%) vs 2295 (9.5%) had additional visits (eFigure 2 in Supplement 1).

**Figure 2.**
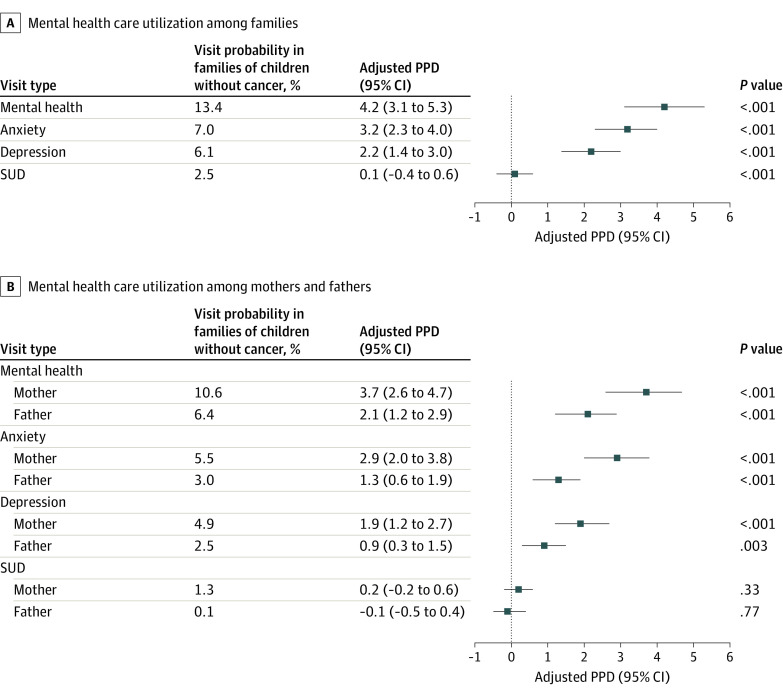
Model-Adjusted Differences in Probability of Mental Health–Related Visits Between Families or Parents of Children With vs Without Cancer Analysis of the Merative MarketScan Commercial Claims Database for families (A) and for mothers (n = 25 654) and fathers (n = 23 607; B). A, Multiple logistic regressions adjusted for age group, CCI score, and mental health history of the household lead as well as rurality of residence, geographic region, health plan type, number of children in the household, child sex, child age at index date, and year of index date. B, Multiple logistic regressions adjusted for age group, CCI score, and mental health history of the mother (or the father) as well as rurality of residence, geographic region, health plan type, number of children in the household, child sex, child age at index date, and year of index date. In both A and B, model-based estimated probability is reported for having visits related to a mental health condition among families or parents of children without cancer, with other covariates held at their observed values. CCI indicates Charlson Comorbidity Index; PPD, percentage point difference; SUD, substance use or related disorder.

In adjusted analysis, we observed that families of children with (vs without) cancer had higher probabilities of 1 or both parents having anxiety-related visits (ME, 3.2 percentage points [95% CI, 2.3 to 4.0]), depression-related visits (ME, 2.2 percentage points [95% CI, 1.4 to 3.0]), and any MH-related visits (ME, 4.2 percentage points [95% CI, 3.1 to 5.3]), respectively, during the year post index (Figure 2A). These values correspond to relative increases of 45.7% (3.2 [ME] divided by 7.0 [adjusted probability of families of children without cancer]), 36.1%, and 31.3% in the probabilities of anxiety-related, depression-related, and any MH-related visits, respectively. A nonsignificant difference in SUD-related visits was observed (ME, 0.1 percentage points [95% CI, −0.4 to 0.6]).

Supplemental analysis of additional visits showed adjusted differences of 1.9 percentage points (95% CI, 1.2 to 2.6) for anxiety-related visits, 1.9 percentage points (95% CI, 1.2 to 2.5) for depression-related visits, and 3.3 percentage points (95% CI, 2.3 to 4.2) for any MH-related visits between families with vs without children diagnosed with cancer (eTable 4 in Supplement 1).

### MH-Related Visits Among Mothers and Fathers

The proportions of MH-related visits were consistently higher for mothers than fathers (617 [14.7%] vs 336 [8.4%] for parents of children with cancer; and 2253 [10.5%] vs 1267 [6.5%] for families of children without cancer; Figure 1B and C). Similar differences between mothers and fathers were observed for anxiety-related and depression-related visits.

In adjusted analyses of fathers, those caring for children with cancer were 43.3% (ME, 1.3 percentage points [95% CI, 0.6 to 1.9]), 36.0% (ME, 0.9 percentage points [95% CI, 0.3 to 1.5]), and 32.8% (ME, 2.1 percentage points [95% CI, 1.2 to 2.9]) more likely to have anxiety-related, depression-related, and any MH-related visits, respectively, than fathers from families of children without cancer (Figure 2B). Differences were also observed among mothers. Mothers caring for children with cancer were 52.7% (ME, 2.9 percentage points [95% CI, 2.0 to 3.8]), 38.8% (ME, 1.9 percentage points [95% CI, 1.2 to 2.7]), and 34.9% (ME, 3.7 percentage points [95% CI, 2.6 to 4.7]) more likely to have anxiety-related, depression-related, and any MH-related visits, respectively, than mothers from families of children without cancer.

### Factors Associated With MH-Related Visits Among Parents of Children With Cancer

Among parents of children with cancer, mothers residing in urban (vs rural) areas were more likely to have any MH-related visits (ME, 3.6 [95% CI, 0.9 to 6.3]) and depression-related visits (ME, 2.2 [95% CI, 0.3 to 4.1]). Mothers enrolled in HMO or PPO plans (vs high-deductible plans) were more likely to have any MH-related visits (HMO: ME, 5.0 [95% CI, 1.1 to 8.9]; or PPO: ME, 4.7 [95% CI, 1.9 to 7.4]) and anxiety-related visits (HMO: ME, 4.8 [95% CI, 1.5 to 8.0]; or PPO: ME, 3.3 [95% CI, 1.2 to 5.5]; Table 2).

**Table 2.** Factors Associated With Probability of Mental Health–Related Visits Among Mothers Caring for Children With Cancer

Characteristic	No. of participants	Visit type^a^								
		Any mental health			Anxiety related			Depression related		
		Unadjusted %^b^	Adjusted PPD (95% CI)^c^	*P* value	Unadjusted % (row)	Adjusted PPD (95% CI)	*P* value	Unadjusted % (row)	Adjusted PPD (95% CI)	*P* value
**Mother**										
Age group, y										
25-34	639	15.6	[Reference]	NA	8.1	[Reference]	NA	7.7	[Reference]	NA
35-44	1713	15.6	−1.0 (−4.5 to 2.5)	.59	9.9	1.8 (−0.7 to 4.3)	.17	7.5	−1.3 (−4.1 to 1.5)	.37
45-54	1551	13.6	−2.6 (−6.5 to 1.4)	.20	8.1	1.1 (−1.8 to 3.9)	.46	6.4	−2.3 (−5.4 to 0.8)	.14
55-64	307	12.4	−3.8 (−8.9 to 1.3)	.15	7.2	0.6 (−3.5 to 4.6)	.78	4.9	−3.4 (−7.2 to 0.4)	.08
CCI score	NA	NA	1.2 (−0.1 to 2.4)	.06	NA	−0.02 (−0.9 to 0.9)	.96	NA	0.9 (0.2 to 1.5)	.01
Residence										
Rural	504	11.7	[Reference]	NA	6.5	[Reference]	NA	5.2	[Reference]	NA
Urban	3706	15.1	3.6 (0.9 to 6.3)	.01	9.0	2.0 (−0.4 to 4.3)	.10	7.2	2.2 (0.3 to 4.1)	.03
US region										
Northeast	985	13.6	[Reference]	NA	8.6	[Reference]	NA	6.4	[Reference]	NA
Midwest or North Central	1005	16.0	2.9 (−0.2 to 6.0)	.06	8.7	0.3 (−2.2 to 2.8)	.81	8.4	2.0 (−0.3 to 4.3)	.09
South	1543	14.2	0.3 (−2.4 to 2.9)	.85	8.9	−0.2 (−2.4 to 2.0)	.84	6.0	−0.2 (−2.0 to 1.7)	.87
West	677	15.2	1.5 (−1.7 to 4.7)	.35	8.6	−0.5 (−3.1 to 2.1)	.69	7.7	1.2 (−1.2 to 3.6)	.33
Health plan type										
High deductible	538	11.0	[Reference]	NA	6.1	[Reference]	NA	5.8	[Reference]	NA
HMO	461	17.1	5.0 (1.1 to 8.9)	.01	11.1	4.8 (1.5 to 8.0)	.004	8.0	1.5 (−1.4 to 4.4)	.30
PPO	2152	15.5	4.7 (1.9 to 7.4)	.001	9.0	3.3 (1.2 to 5.5)	.003	7.2	1.5 (−0.7 to 3.7)	.17
Other	1059	13.8	3.6 (0.5 to 6.7)	.02	8.5	3.2 (0.7 to 5.7)	.01	6.4	0.6 (−1.8 to 3.0)	.65
No. of children										
1	577	16.6	[Reference]	NA	9.0	[Reference]	NA	8.1	[Reference]	NA
2	1875	14.8	−1.5 (−4.9 to 1.9)	.39	8.4	−1.2 (−3.9 to 1.5)	.40	7.0	−0.8 (−3.3 to 1.7)	.52
≥3	1758	13.9	−2.7 (−6.2 to 0.7)	.11	9.0	−0.7 (−3.5 to 2.1)	.62	6.5	−1.6 (−4.1 to 0.8)	.20
**Child**										
Sex										
Male	2469	14.7	[Reference]	NA	9.1	[Reference]	NA	6.9	[Reference]	NA
Female	1741	14.5	0.4 (−1.7 to 2.4)	.73	8.3	−0.2 (−1.9 to 1.5)	.83	7.0	0.1 (−1.3 to 1.6)	.85
Age at index date, y										
0-4	753	15.5	[Reference]	NA	9.7	[Reference]	NA	6.9	[Reference]	NA
5-14	1452	15.2	−0.6 (−3.9 to 2.7)	.73	10.0	−0.8 (−3.7 to 2.2)	.60	7.4	0.6 (−1.7 to 2.8)	.62
15-21	2005	14.0	−1.0 (−4.5 to 2.6)	.60	7.5	−3.0 (−6.0 to −0.04)	.047	6.6	0.5 (−2.0 to 3.1)	.69
Cancer type										
Hematologic	2047	13.0	[Reference]	NA	7.7	[Reference]	NA	6.1	[Reference]	NA
Bone and soft tissue	548	17.2	2.8 (−0.5 to 6.1)	.10	8.6	0.3 (−2.3 to 2.9)	.84	9.5	3.0 (0.3 to 5.7)	.03
CNS	972	16.6	2.8 (−0.04 to 5.7)	.05	10.8	2.4 (0.004 to 4.8)	.05	6.9	1.1 (−0.9 to 3.1)	.28
Gonadal	430	14.9	2.6 (−1.2 to 6.3)	.19	9.3	3.8 (0.3 to 7.3)	.03	6.7	1.1 (−1.7 to 3.8)	.44
Multiple types	213	14.6	0.5 (−4.2 to 5.2)	.83	8.9	1.1 (−2.7 to 4.9)	.58	8.9	2.1 (−1.4 to 5.6)	.24
Treatment type										
Surgery only	903	14.1	[Reference]	NA	8.1	[Reference]	NA	6.9	[Reference]	NA
Any HSCT	236	14.8	2.5 (−2.8 to 7.9)	.35	8.9	0.8 (−3.2 to 4.9)	.68	8.1	2.9 (−1.4 to 7.1)	.19
Any radiation (no HSCT)	1172	16.1	1.9 (−1.0 to 4.9)	.21	9.6	1.9 (−0.5 to 4.3)	.13	7.1	0.3 (−1.8 to 2.3)	.78
Any chemotherapy (no HSCT or radiation)	1899	14.0	−0.1 (−2.9 to 2.8)	.97	8.5	0.4 (−1.9 to 2.8)	.71	6.7	0.4 (−1.7 to 2.4)	.73

Abbreviations: CCI, Charlson Comorbidity Index; CNS, central nervous system; HMO, health maintenance organization; HSCT, hematopoietic stem cell transplantation; NA, not applicable; PPD, percentage point difference; PPO, preferred provider organization.

^a^ Analysis of Merative MarketScan Commercial Claims Database. Multiple logistic regression models controlled for parent mental health history and year of index date.

^b^ Unadjusted row percentages.

^c^ Adjusted PPDs (ie, marginal effects) were generated based on estimated probabilities of outcomes for families of children with vs without cancer, with other covariates calculated at their observed values; 95% CIs were generated using the delta method.

Additionally, mothers caring for children diagnosed with gonadal (vs hematologic) cancer had an increase of 3.8 percentage points (95% CI, 0.3 to 7.3) in the probability of anxiety-related visits. Mothers of children diagnosed with bone and soft tissue (vs hematologic) cancers had an increase of 3.0 percentage points (95% CI, 0.3 to 5.7) in the probability of depression-related visits (Table 2). We did not find factors associated with SUD-related visits among mothers or with anxiety-related, depression-related, SUD-related, and any MH-related visits among fathers (Table 3 and eTable 5 in Supplement 1).

**Table 3.** Factors Associated With the Probability of Mental Health–Related Visits Among Fathers Caring for Children With Cancer

Characteristic	No. of participants	Visit type^a^								
		Any mental health			Anxiety related			Depression related		
		Unadjusted %^b^	Adjusted PPD (95% CI)^c^	*P* value	Unadjusted %	Adjusted PPD (95% CI)	*P* value	Unadjusted %	Adjusted PPD (95% CI)	*P* value
**Father**										
Age group, y										
25-34	394	7.4	[Reference]	NA	3.8	[Reference]	NA	2.5	[Reference]	NA
35-44	1439	9.2	1.5 (−1.4 to 4.4)	.31	5.0	0.5 (−1.9 to 2.9)	.67	3.4	1.1 (−0.6 to 2.7)	.19
45-54	1663	7.8	−0.2 (−3.2 to 2.9)	.92	3.7	−0.9 (−3.3 to 1.6)	.48	3.5	1.2 (−0.7 to 3.0)	.22
55-64	520	8.7	0.7 (−3.1 to 4.5)	.72	4.4	0.04 (−3.0 to 3.0)	.98	3.5	1.2 (−1.2 to 3.5)	.33
CCI score	NA	NA	−0.4 (−1.6 to 0.8)	.55	NA	−0.1 (−0.8 to 0.7)	.89	NA	0.2 (−0.8 to 1.1)	.75
Residence										
Rural	483	9.5	[Reference]	NA	4.8	[Reference]	NA	3.3	[Reference]	NA
Urban	3533	8.2	−1.7 (−4.5 to 1.0)	.22	4.2	−0.9 (−3.1 to 1.2)	.39	3.4	−0.1 (−2.0 to 1.8)	.92
US region										
Northeast	958	9.7	[Reference]	NA	5.3	[Reference]	NA	4.7	[Reference]	NA
Midwest or North Central	988	9.2	−0.1 (−2.5 to 2.4)	.96	4.7	−0.6 (−2.5 to 1.3)	.51	3.2	−1.1 (−2.7 to 0.6)	.21
South	1422	7.9	−1.5 (−3.7 to 0.7)	.17	3.7	−1.6 (−3.3 to 0.2)	.08	2.8	−1.4 (−2.8 to 0.1)	.08
West	648	6.2	−2.8 (−5.3 to −0.3)	.03	3.2	−1.8 (−3.7 to 0.2)	.07	2.8	−1.5 (−3.3 to 0.3)	.10
Health plan type										
High deductible	519	6.2	[Reference]	NA	3.5	[Reference]	NA	2.5	[Reference]	NA
HMO	430	9.8	2.8 (−0.6 to 6.2)	.11	5.3	2.0 (−0.6 to 4.5)	.13	3.5	0.6 (−1.5 to 2.8)	.55
PPO	2061	8.4	1.5 (−0.9 to 3.9)	.23	4.2	1.0 (−0.7 to 2.8)	.26	3.4	0.7 (−0.9 to 2.3)	.42
Other	1006	8.7	2.0 (−0.7 to 4.7)	.14	4.3	0.6 (−1.3 to 2.5)	.53	3.6	0.7 (−1.0 to 2.5)	.42
No. of children										
1	499	10.6	[Reference]	NA	4.2	[Reference]	NA	4.8	[Reference]	NA
2	1789	8.7	−1.1 (−3.9 to 1.7)	.45	4.6	0.7 (−1.2 to 2.5)	.47	3.6	−0.7 (−2.8 to 1.3)	.49
≥3	1728	7.3	−2.7 (−5.4 to 0.04)	.05	3.9	−0.1 (−1.9 to 1.7)	.91	2.7	−1.6 (−3.6 to 0.5)	.13
**Child**										
Sex										
Male	2345	8.6	[Reference]	NA	4.2	[Reference]	NA	3.8	[Reference]	NA
Female	1671	8.0	−0.4 (−2.0 to 1.2)	.64	4.4	−0.1 (−1.3 to 1.1)	.90	2.8	−0.7 (−1.7 to 0.4)	.23
Age at index date, y										
0-4	731	8.6	[Reference]	NA	4.2	[Reference]	NA	3.7	[Reference]	NA
5-14	1387	8.5	−0.1 (−2.6 to 2.4)	.94	4.7	0.6 (−1.3 to 2.4)	.54	3.3	−1.0 (−2.9 to 0.9)	.32
15-21	1898	8.2	−0.9 (−3.6 to 1.9)	.54	4.0	−0.02 (−2.0 to 2.0)	.99	3.3	−1.5 (−3.7 to 0.7)	.18
Cancer type										
Hematologic	1945	8.6	[Reference]	NA	4.5	[Reference]	NA	3.3	[Reference]	NA
Bone and soft tissue	525	8.6	0.1 (−2.6 to 2.8)	.96	4.6	−0.3 (−2.3 to 1.6)	.76	3.6	0.8 (−1.2 to 2.8)	.42
CNS	933	7.5	−1.4 (−3.5 to 0.7)	.18	4.2	−0.8 (−2.4 to 0.9)	.35	3.1	−0.02 (−1.4 to 1.4)	.98
Gonadal	411	8.5	0.7 (−2.5 to 3.8)	.68	3.4	−0.6 (−2.9 to 1.6)	.58	3.6	0.6 (−1.5 to 2.6)	.59
Multiple types	202	8.9	0.6 (−3.4 to 4.7)	.75	3.0	−1.2 (−3.7 to 1.3)	.34	3.5	0.1 (−2.4 to 2.6)	.91
Treatment type										
Surgery only	868	7.6	[Reference]	NA	3.6	[Reference]	NA	3.3	[Reference]	NA
Any HSCT	213	8.0	1.2 (−2.7 to 5.1)	.54	2.8	−0.4 (−3.0 to 2.3)	.79	4.7	1.8 (−1.1 to 4.7)	.21
Any radiation (no HSCT)	1092	8.8	1.4 (−1.0 to 3.8)	.25	4.7	1.1 (−0.7 to 2.9)	.23	3.3	0.3 (−1.3 to 1.9)	.72
Any chemotherapy (no HSCT or radiation)	1843	8.5	0.9 (−1.3 to 3.2)	.42	4.5	0.7 (−1.0 to 2.4)	.40	3.3	0.3 (−1.1 to 1.7)	.71

Abbreviations: CCI, Charlson Comorbidity Index; CNS, central nervous system; HMO, health maintenance organization; HSCT, hematopoietic stem cell transplantation; PPD, percentage point difference; PPO, preferred provider organization.

^a^ Analysis of Merative MarketScan Commercial Claims Database. Multiple logistic regression models controlled for parent mental health history and year of index date.

^b^ Unadjusted row percentages.

^c^ Adjusted PPDs (ie, marginal effects) were generated based on estimated probabilities of outcomes for families of children with vs without cancer, with other covariates calculated at their observed values; 95% CIs were generated using the delta method.

## Discussion

To our knowledge, this study provides the first evidence on utilization of MH services among parents of children diagnosed with cancer in the US. We observed that parents of children with cancer had statistically significantly higher probabilities of MH-related visits, particularly visits related to anxiety and depression, compared with the general parent population. This difference, although observed among both fathers and mothers, was greater in magnitude among mothers. Among mothers caring for children diagnosed with cancer, enrollment in high-deductible plans and rural residence were associated with decreased probabilities of MH-related visits.

Our study estimated that 18.1% of parents caring for children with cancer had any MH visits; among these, 10.6% had an anxiety-related visit and 8.4% had a depression-related visit. Several prior studies based on qualitative interviews or cross-sectional surveys showed that 9% to 74% and 5% to 46% of parents of children with cancers reported a clinically relevant level of anxiety and depression symptoms, respectively.^7,8,9^ Our analysis differs from previous studies in terms of study design, MH care outcomes, and geographic representation. Particularly, the use of claims data allowed us to accurately measure realized MH care. Notably, one prior claim-based analysis similarly showed a higher prevalence of MH diagnoses among parents caring for children with severe health conditions, including cancer, compared with those caring for children without severe conditions.^38^ That study was limited to a single private insurance company and lacked measures of types of MH diagnosis and cancer-related characteristics. Our analysis adds to the literature by leveraging nationwide commercial claims from multiple insurers and including a comprehensive set of measures for MH service utilization, sociodemographic factors, and cancer-related characteristics.

There are several possible reasons for the increased probability of MH visits in parents caring for children with cancer. First, in addition to witnessing the symptoms and suffering experienced by their child, the uncertainty of prognosis and survival can pose substantial fear and worry to parents, which can worsen existing MH symptoms or trigger new MH symptoms.^10^ Caring for children diagnosed with bone or soft tissue and gonadal cancers was associated with higher probabilities of depression-related and anxiety-related visits among mothers, respectively, compared with caring for children with hematologic cancers. This difference may reflect poorer prognosis, more severe pain, or fertility concerns in nonhematologic cancers that could contribute to higher MH needs.^39,40^

Second, the cancer treatment process demands an overwhelming amount of time and effort from parents, including coordinating medical appointments, administering medications, communicating with health care providers and insurance companies, assisting children’s daily activities, and providing companionship.^41,42^ Consequently, parents may experience substantial disruptions in family and social interactions and face challenges due to absence from work and other competing responsibilities, which can trigger MH problems.^18^ Third, the financial stress of covering medical expenses of cancer treatment could further exacerbate the negative impact of pediatric cancer diagnoses on parent psychological health.^18,43^

It is worth noting that our measures of MH care visits reflect a complex concept involving both psychological needs for MH treatments and parents’ ability to access these treatments. For example, the observed increase in utilization among parents of children with cancer may have benefited from care standards recommending psychosocial assessment and interventions, which could improve access to MH referral and treatment for caregivers.^16^ Conversely, financial and time constraints, while contributing to higher MH care needs, may hinder parents from seeking MH services, resulting in unrealized MH care needs not captured by claims data. This may partially explain the differences between our estimates based on claims data and previous estimates using self-report data.^44,45^ In our data, mothers enrolled in high-deductible plans or living in rural areas were much less likely to have an MH visit; this may reflect the parent subgroups with higher MH needs due to more constraints but poorer accessibility to MH care resources with high-deductible plans and rural residency.

Our finding that MH care utilization differed between mothers and fathers is consistent with sex differences observed among the general adult population.^46^ Sex differences in MH service utilization might be explained partially by biological differences^47^ in neural response to psychological stress, reactivity, and subjective report of stress.^48,49^ Societal expectations may also affect fathers’ expression and reports of stress as well as coping strategies.^50,51^ Another potential explanation is that mothers are more often the primary caregiver, and they experience more stress during their child’s cancer diagnosis and treatment process.^52^ This explanation is consistent with a prior study that compared 2 groups of families with fathers and mothers, respectively, as the primary caregiver, in which no sex difference in psychological distress was detected.^9^

Unlike depression and anxiety, the prevalence of SUD in the present study was much lower and comparable between families of children with vs without cancer. Because the neurobiological process of SUD takes time to develop and manifest,^53^ there should not be a significant short-term impact of a pediatric cancer diagnosis on parental SUD. In addition, SUD may be underdiagnosed due to social stigmatization and because diagnosis codes recorded in claims are primarily for billing purposes^54,55^; however, such underestimation would not differentially affect parents of children with vs without cancer.

### Limitations

This study has several limitations. First, administrative data are generated for billing purposes and therefore restricted. We had no data on self-paid or other insurer-paid visits or nonbilled psychosocial services provided to caregivers in pediatric oncology settings. Thus, we may have underestimated the prevalence of MH visits. Additionally, we could not capture unmet needs among parents (ie, those who had MH needs but did not seek MH services), and we lacked data on enrollee race and ethnicity, income, and cancer stage, which may be important factors associated with MH care needs and merit future investigation. The observed rural-urban differences in MH care utilization among mothers may be associated with the differential billing behaviors of providers in rural areas, a factor we were unable to measure.^56^

Second, because MarketScan data consist of a nationwide convenience sample covered by medium to large employers, our findings may not generalize to publicly insured or uninsured parents. The higher prevalence of material hardship among families with public or no insurance may exaggerate the stress response and subsequent MH care utilization when facing their child’s cancer diagnosis.^57^ Third, because our sample did not include pediatric cancer types (eg, Wilms cancer) that lack validated claims-based algorithms, our findings may not generalize to other pediatric cancers.

Finally, to minimize sample attrition from discontinued insurance enrollment over time, our analysis focused on MH care utilization within 1 year following children’s cancer diagnosis. Future research could examine longer trajectories of psychological stress and MH care utilization.

## Conclusions

In this cohort study of privately insured families, parents caring for children diagnosed with cancer had greater utilization of MH services than other parents. These findings underline the importance of multilevel interventions—such as providing MH screening, counseling, and timely support and ensuring comprehensive insurance coverage and paid medical leave—to better meet the MH needs of these parents. Increased attention might be warranted for parents with lower MH service utilization, including mothers enrolled in high-deductible plans and those living in rural areas.

## Notes

Supplement 1. **eTable 1.** Agency for Healthcare Research and Quality Clinical Classification Software (CCS) Categories for Cancer Diagnoses **eFigure 1.** Sample Derivation Flowchart **eTable 2.** Sample Characteristics Comparing Families Included in the Final Analysis and the Excluded Families **eMethods.** Detailed Description of the Matching Process **eTable 3.** Mental Health Diagnosis Codes **eFigure 2.** Unadjusted Probability of Mental Health–Related Visits During the Year Post Index Date Among Families or Parents Caring for Children With vs Without Cancer **eTable 4.** Model-Adjusted Differences in the Probability of Having Additional Visits Related to a Mental Health Condition Between Families or Parents of Children With vs Without Cancer **eTable 5.** Factors Associated With the Likelihood of SUD-Related Visits Among Parents Caring for Children With Cancer
eReferences

Supplement 2.
Data Sharing Statement
