# Supplementary material for: Mental Health Care Utilization Among Parents of Children With Cancer
Source: JAMA Netw Open. 2024 Apr 2;7(4):e244531. doi: 10.1001/jamanetworkopen.2024.4531 (PMC10988353; doi:10.1001/jamanetworkopen.2024.4531)
Supplement: Supplement 1. — eTable 1. Agency for Healthcare Research and Quality Clinical Classification Software (CCS) Categories for Cancer Diagnoses eFigure 1. Sample Derivation Flowchart eTable 2. Sample Characteristics Comparing Families Included in the Final Analysis and the Excluded Families eMethods. Detailed Description of the Matching Process eTable 3. Mental Health Diagnosis Codes eFigure 2. Unadjusted Probability of Mental Health–Related Visits During the Year Post Index Date Among Families or Parents Caring for Children With vs Without Cancer eTable 4. Model-Adjusted Differences in the Probability of Having Additional Visits Related to a Mental Health Condition Between Families or Parents of Children With vs Without Cancer eTable 5. Factors Associated With the Likelihood of SUD-Related Visits Among Parents Caring for Children With Cancer eReferences [file jamanetwopen-e244531-s001.pdf]

Supplementary Online Content

Hu X, Grosse SD, Han X, Marchak JG, Ji X. Mental health care utilization among parents of children with cancer. *JAMA Netw Open*. 2024;7(4):e244531. doi:10.1001/jamanetworkopen.2024.4531

eTable 1. Agency for Healthcare Research and Quality Clinical Classification Software (CCS) Categories for Cancer Diagnoses

eFigure 1. Sample Derivation Flowchart

eTable 2. Sample Characteristics Comparing Families Included in the Final Analysis and the Excluded Families

eMethods. Detailed Description of the Matching Process

eTable 3. Mental Health Diagnosis Codes

eFigure 2. Unadjusted Probability of Mental Health–Related Visits During the Year Post Index Date Among Families or Parents Caring for Children With vs Without Cancer

eTable 4. Model-Adjusted Differences in the Probability of Having Additional Visits Related to a Mental Health Condition Between Families or Parents of Children With vs Without Cancer

eTable 5. Factors Associated With the Likelihood of SUD-Related Visits Among Parents Caring for Children With Cancer

eReferences

This supplementary material has been provided by the authors to give readers additional information about their work.

© 2024 Hu X et al. *JAMA Network Open.*

© 2024 Hu X et al. *JAMA Network Open.*

eTable 1. Agency for Healthcare Research and Quality Clinical Classification Software (CCS) Categories for Cancer Diagnoses

CCS Categories	Cancer Types
37, 38, 39	Hematologic cancers (leukemia, lymphoma)
35	Central nervous system
21	Bone or connective tissue
27, 30	Gonadal Cancer

Notes: Detailed ICD codes for each CCS category can be downloaded from:
https://www.hcup-us.ahrq.gov/toolssoftware/ccs/ccs.jsp
https://www.hcup-us.ahrq.gov/toolssoftware/ccs10/ccs10.jsp

eFigure 1. Sample Derivation Flowchart

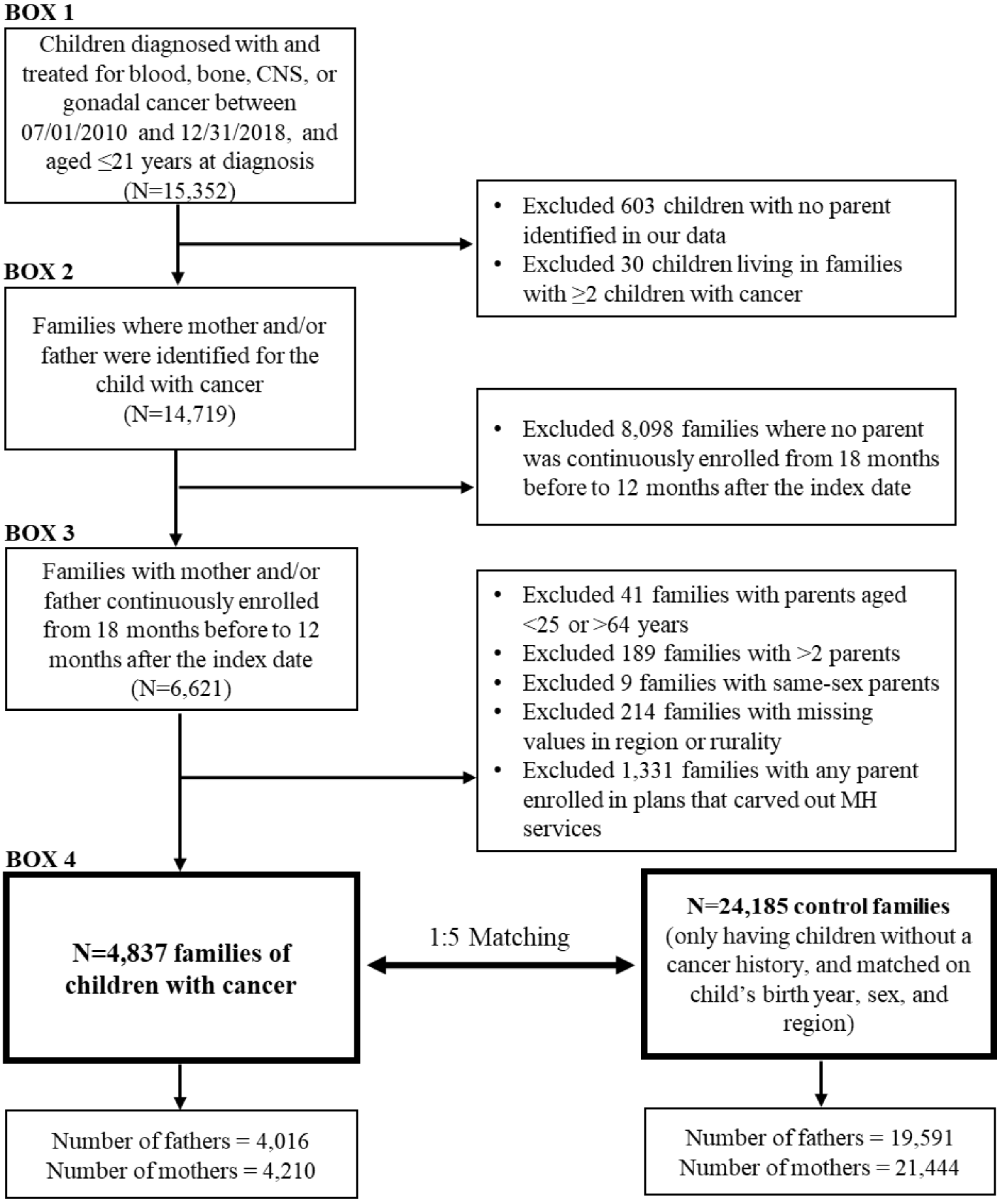

Notes: This study focused on children diagnosed with cancer between 07/01/2010 and 12/31/2018 to ensure each child had an 18-month window before diagnosis and a 12-month window following diagnosis for assessment, since the MarketScan data available to the study team were between 2009 and 2019.

© 2024 Hu X et al. *JAMA Network Open.*

eTable 2. Sample Characteristics Comparing Families Included in the Final Analysis and the Excluded Families

Characteristics	Families caring for children with cancer that were included in the final analysis (n=4,837)	Excluded Families (n=9,882)[a]
	No. (%)[b]	No. (%)[b]
Parent insurance enrollment status		
Mother enrollment only	821 (17.0)	1411 (14.3)
Father enrollment only	627 (13.0)	925 (9.4)
Both mother and father	3389 (70.1)	7534 (76.2)
Parent age[c], mean (standard deviation)	44.8 (8.0)	43.4 (8.4)
Rurality of residence		
Rural	585 (12.1)	1293 (13.1)
Urban	4252 (87.9)	8494 (86.0)
Missing	0 (0.0)	95 (1.0)
US geographic region[d]		
Northeast	1109 (22.9)	1774 (18.0)
Midwest/North Central	1156 (23.9)	2230 (22.6)
South	1799 (37.2)	3518 (35.6)
West	773 (16.0)	2078 (21.0)
Missing	0 (0.0)	282 (2.9)
Health plan type		
High deductible	616 (12.7)	1004 (10.2)
HMO	526 (10.9)	774 (7.8)
PPO	2473 (51.1)	5798 (58.7)
Other[e]	1222 (25.3)	2306 (23.3)
Number of children in the household		
1	702 (14.5)	1643 (16.6)
2	2137 (44.2)	4243 (42.9)
3 or more	1998 (41.3)	3996 (40.4)
Child's age at index date[f]		
0-4 years	860 (17.8)	1860 (18.8)
5-14 years	1670 (34.5)	3985 (40.3)
15-21 years	2307 (47.7)	4037 (40.9)
Child's sex		
Male	2837 (58.7)	5777 (58.5)
Female	2000 (41.3)	4105 (41.5)
Year of the child's index date[f]		
2010	362 (7.5)	611 (6.2)
2011	796 (16.5)	1796 (18.2)
2012	676 (14.0)	1694 (17.1)
2013	646 (13.4)	1351 (13.7)
2014	521 (10.8)	1456 (14.7)
2015	577 (11.9)	656 (6.6)
2016	514 (10.6)	714 (7.2)
2017	389 (8.0)	730 (7.4)
2018	356 (7.4)	874 (8.8)
Child's cancer types		
Hematologic cancer	2350 (48.6)	5388 (54.5)
Bone and soft tissue cancers	627 (13.0)	1245 (12.6)
CNS cancers	1119 (23.1)	2170 (22.0)
Gonadal cancers	500 (10.3)	733 (7.4)
Multiple types of cancers	241 (5.0)	346 (3.5)
Child's cancer treatment modality[g]		

© 2024 Hu X et al. *JAMA Network Open.*

Any HSCT	271 (5.6)	408 (4.1)
Any radiation (no HSCT)	1334 (27.6)	2233 (22.6)
Any chemotherapy (no HSCT or radiation)	2199 (45.5)	5525 (55.9)
Surgery only	1033 (21.4)	1716 (17.4)

Notes: Authors' analysis of the Merative™ MarketScan® Commercial Database.
Abbreviations: HMO – health maintenance organization. PPO – preferred provider organization. SD – standard deviation. CNS – central nervous system. HSCT – hematopoietic stem cell transplantation.
[a] Excluded families consist of those excluded between BOX 2 and BOX 4 described in eFigure 1.
[b] Column percentage reported.
[c] For family-level analysis, these characteristics (i.e., parent age group) refer to the characteristics of the household lead.
[d] Region follows the categorization defined by the US Census Bureau: https://www2.census.gov/geo/pdfs/maps-data/maps/reference/us_regdiv.pdf.
[e] Other insurance types include basic/major medical plan, comprehensive plan, exclusive provider organization, non-capitated point-of-service plan, and capitated or partially-capitated point-of-service plan.
[f] Index date for a child with cancer was defined as the date of the first healthcare claim with a cancer diagnosis.
[g] To measure children's treatment modality, we created a mutually exclusive categorical variable that follows a hierarchical coding strategy to classify children with cancer into the following four groups who: 1) received any HSCT; 2) did not receive any HSCT, but received any radiation therapy; 3) did not receive any HSCT or radiation, but received any chemotherapy; and 4) did not receive any HSCT, radiation, or chemotherapy, but received surgery only.

© 2024 Hu X et al. *JAMA Network Open.*

eMethods. Detailed Description of the Matching Process

Consistent with prior research,[1–3] the process of matching non-cancer families to families of children with cancer included the following steps:

Step 1: Identify eligible children without cancer, based on year of birth, sex, and region strata.

We first identified unique combinations (i.e., strata) of year of birth, sex, and geographic region among all eligible children with cancer. Then, for each stratum, we identified all children without cancer in the MarketScan Commercial Claims and Encounters Database who had the same year of birth, sex, and geographic region. Notably, children without cancer were defined as children who did not have any inpatient or outpatient healthcare claim with International Classification of Disease (ICD) codes that fell within cancer-related Clinical Classification Software (CCS) categories during the entire study period, as done in prior research.[2,4]

Step 2: Within each stratum, randomly match children without cancer to children with cancer.

Within each stratum (with the same year of birth, sex, and geographic region), there were two scenarios. If there was a single child with cancer in the stratum, all children without cancer identified in Step 1 in that stratum would be included to be matched to this child with cancer. If there were multiple children with cancer in the same stratum, we randomly selected approximately the same number of children without cancer, without replacement, to be matched to each child with cancer. More specifically, if in a stratum there were 10 children with cancer and 312 children without cancer identified in Step 1, we would randomly select approximately 31 children without cancer (without replacement) to be matched to each of the 10 children with cancer.

Step 3: Assign each non-cancer child a pseudo "index date" and assess continuous enrollment for his/her parent(s) from 18 months before through 12 months after the "index date."

Rationale of this step: For the parents caring for children with cancer in our sample, we required continuous enrollment in their health plan from 18 months before through 12 months after their "index date." We applied a similar continuous enrollment criterion to the parents who did not have a child with cancer. This criterion allows us to capture a relatively complete picture of parents' healthcare utilization, including mental healthcare utilization.

Algorithm: We first assigned the index date of the child with cancer to his/her matched children without cancer as a pseudo "index date." We then identified the parents of these matched children using family ID and the information indicating parents' relationship with the child ("Children"/ "Dependent" of the "Employee"). Next, we restricted to parents with continuous insurance enrollment from 18 months before through 12 months after their pseudo "index date." Notably, each child with cancer and his/her matched children without cancer had the same year of birth and "index date," and thus, they were at the same age in the year of their "index date."

We further excluded (a) families with parents aged <25 years or >64 years, (b) families with >2 parents or same-sex parents, (c) families missing information on geographic region or rurality, and (d) families with any parent enrolled in plans that carved out mental health services. These exclusion criteria were also applied to families of children with cancer.

© 2024 Hu X et al. *JAMA Network Open.*

Step 4: Randomly select five non-cancer families for each family of children with cancer.

Using the MarketScan Commercial Claims and Encounters Database, each family that had a child with cancer was able to be matched to a relatively large number (ranging from 59 to 17,834) of non-cancer families using the algorithm detailed in Steps 1-3 above. Lastly, for each family that had a child with cancer, we randomly selected five matched non-cancer families to be included in our analytic sample.

© 2024 Hu X et al. *JAMA Network Open.*

eTable 3. Mental Health Diagnosis Codes

Condition Category	Coding System	ICD Diagnosis Code
Anxiety	ICD-9 codes	293.84, 300.0x, 300.1x, 300.2x, 300.3, 300.5, 300.6, 300.7, 300.8x, 300.9, 308.x, 309.81, 313.0, 313.1, 313.21, 313.22 313.3, 313.82, 313.83
	ICD-10 codes	F06.4, F40.x, F41.x, F42x, F43.0, F43.1x, F43.21, F43.23, F44.0, F44.1, F44.2, F44.4, F44.5, F44.6, F44.7, F44.8x, F44.9, F45.0, F45.1, F45.2, F45.8, F45.9, F48.1, F48.8, F48.9, F68.11, F68.13, F68.8, F93.8, F99, R45.2-R45.7, R45.81-R45.84
Depression	ICD-9 codes	296.2x, 296.3x, 300.4, 311
	ICD-10 codes	F32.0-F32.5, F32.8x, F32.9, F33.0, F33.1, F33.2, F33.3, F33.4x, F33.8, F33.9, F34.1
Substance Use and Related Disorders	ICD-9 codes	291.x, 292.0, 292.1x, 292.2, 292.8x, 292.9, 303.0x, 303.9x, 304.x, 305.x, 357.5, 425.5, 535.3x, 571.0, 571.1, 571.2, 571.3, 648.3x, 655.x, 760.71-760.75, 965.0x, 779.5, 970.1, 980.0, E85.00-E85.02, E93.50-E93.52, E94.01, V65.42
	ICD-10 codes	F10-F19, F55.x, G62.1, HZ81ZZZ, HZ86ZZZ, HZ95ZZZ, HZ84ZZZ, HZ85ZZZ, HZ91ZZZ, HZ94ZZZ, HZ96ZZZ, I42.6, K29.2x, K70.0, K70.1x, K70.2, K703x, K70.40, K70.9, O35.5XXx, O99.31x, O99.32x, P04.3, P04.41, P04.49, P96.1, P96.2, Q86.0, T40.0X1x, T40.0X2x, T40.0X3x, T40.0X4x, T40.0X5x, T40.0X6x, T40.1X1x, T40.1X2x, T40.1X3x, T40.1X4x, T40.1X5x, T40.2X1x, T40.2X2x, T40.2X3x, T40.2X4x, T40.2X5x, T40.3X1x, T40.3X2x, T40.3X3x, T40.3X4x, T40.3X5x, T40.4X1x, T40.4X2x, T40.4X3x, T40.4X4x, T40.4X5x, T40.5X1x, T40.5X3x, T40.5X4x, T40.5X5x, T40.5X6x, T40.601x, T40.602x, T40.603x, T40.604x, T40.605x, T40.691x, T40.692x, T40.693x, T40.694x, T40.695x, T40.7X1x, T40.7X2x, T40.7X3x, T40.7X4x, T40.7X5x, T40.7X6x, T40.8X1x, T40.8X2x, T40.8X3x, T40.8X4x, T40.8X5x, T40.901x, T40.902x, T40.903x, T40.904x, T40.905x, T40.906x, T40.991x, T40.992x, T40.993x, T40.994x, T40.995x, T40.996x, T510X1A, T510X2A, T510X3A, T510X4A, T511X1A, T511X2A, T511X3A, T511X4A, T512X1A, T512X2A, T512X3A, T512X4A, T513X1A, T513X2A, T513X3A, T513X4A, T518X1A, T518X2A, T518X3A, T518X4A, T519X1A, T519X2A, T519X3A, T519X4A

© 2024 Hu X et al. *JAMA Network Open.*

eFigure 2. Unadjusted Probability of Mental Health–Related Visits During the Year Post Index Date Among Families or Parents Caring for Children With vs Without Cancer[a]

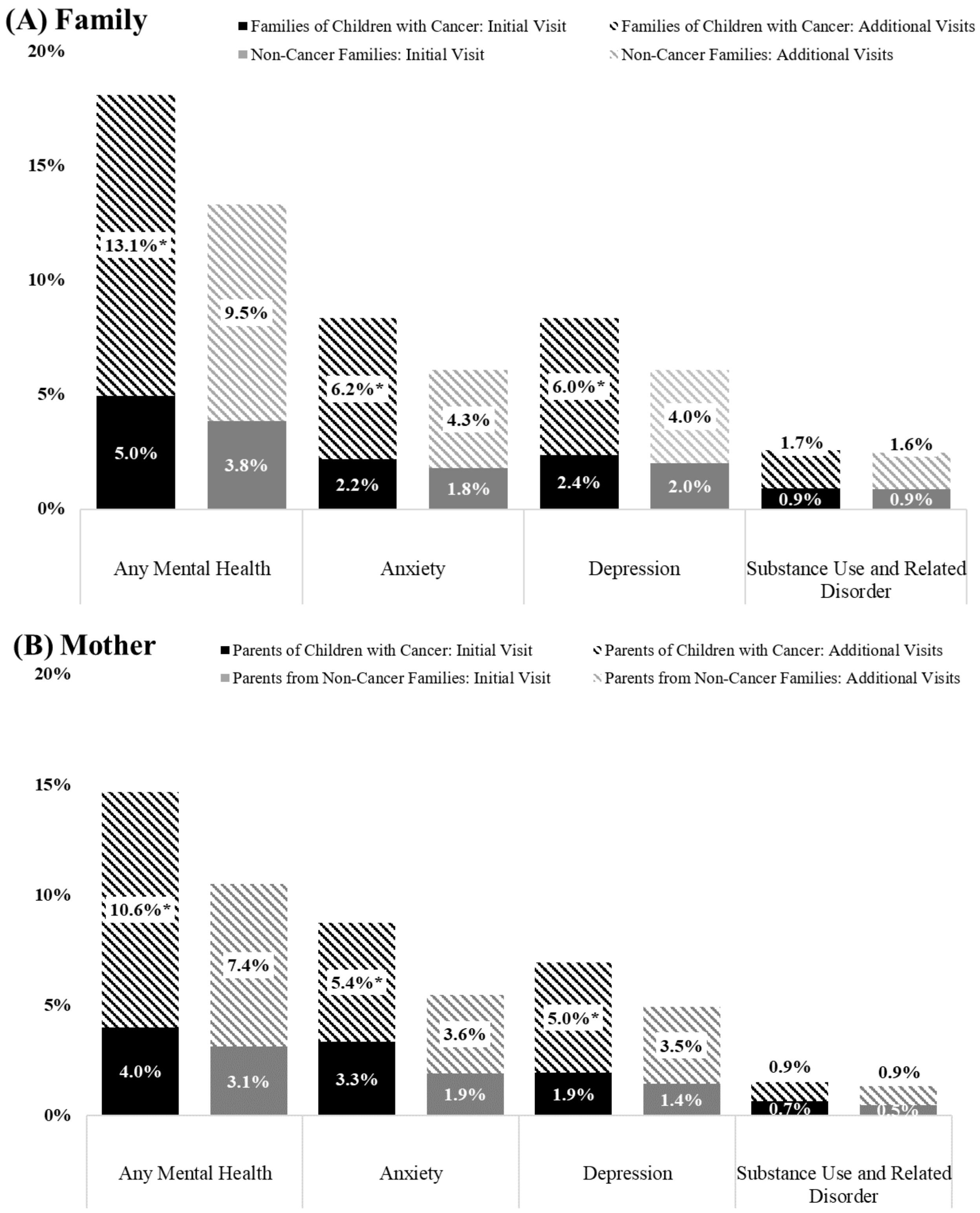

© 2024 Hu X et al. *JAMA Network Open.*

(C) Father

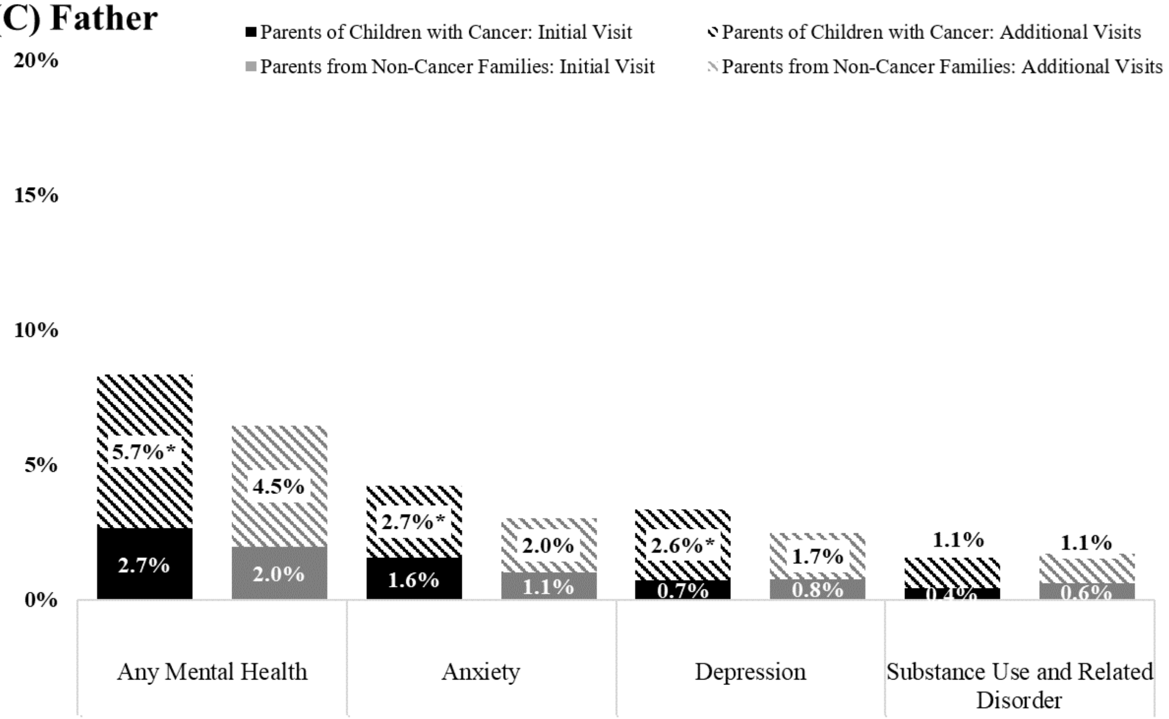

Notes: Authors' analysis of the Merative™ MarketScan® Commercial Database.
[a]Based on the frequencies of parents' visits related a mental health (MH) condition, we conducted a supplemental analysis where we classified parents into those who had 1) no visit, 2) an initial visit only, or 3) visits in addition to the initial visit (i.e., additional visits) during the year following the index date. Notably, to define a MH condition, our main analysis had required at least two outpatient claims, on distinct dates, and/or at least one inpatient claim with the corresponding ICD-9-CM or ICD-10-CM codes during the year following the index date. Accordingly, in this supplemental analysis, parents having an initial visit only refer to those who had only one inpatient claim, or only two outpatient claims, for a specific MH condition (i.e., only satisfy the minimum requirement to be classified as having an MH-related visit). Likewise, patients having additional visits refer to those who had more than one inpatient claim and/or more than two outpatient claims for a specific MH condition (i.e., beyond the minimum requirement).
**P*<0.05 in the comparison of the percentage with additional visits between families of children with cancer and non-cancer families.

© 2024 Hu X et al. *JAMA Network Open.*

eTable 4. Model-Adjusted Differences in the Probability of Having Additional Visits Related to a Mental Health Condition Between Families or Parents of Children With vs Without Cancer

Characteristics	Additional visits	
	Adjusted percentage point difference (95% CI)	*P*
Among Families [a]		
Mental health visits	3.3 (2.3, 4.2)	<.001
Anxiety-related visits	1.9 (1.2, 2.6)	<.001
Depression-related visits	1.9 (1.2, 2.5)	<.001
SUD-related visits	0.01 (-0.36, 0.39)	0.94
Among Mothers [b]		
Mental health visits	3.0 (2.1, 3.8)	<.001
Anxiety-related visits	1.7 (1.0, 2.4)	<.001
Depression-related visits	1.5 (0.8, 2.1)	<.001
SUD-related visits	-0.02 (-0.03, 0.03)	0.88
Among Fathers [b]		
Mental health visits	1.3 (0.6, 2.1)	<.001
Anxiety-related visits	0.7 (0.2, 1.3)	0.006
Depression-related visits	1.0 (0.5, 1.5)	<.001
SUD-related visits	0.03 (-0.33, 0.39)	0.87

Notes: The authors' analysis of the Merative™ MarketScan® Commercial Database.
Abbreviations: SUD – substance use and related disorder. CI – confidence interval.
[a] Estimates generated using Stata "margins" command after the generalized ordered logistic regression, controlling for the age group, Charlson Comorbidity Index, and mental health history of the household lead, rurality of residence, geographic region, health plan type, number of children in the household, child's sex, child's age at index date, and year of index date.
[b] Estimates generated using Stata "margins" command after the generalized ordered logistic regression, controlling for the age group, Charlson Comorbidity Index, and mental health history of the mother (or the father), rurality of residence, geographic region, health plan type, number of children in the household, child's sex, child's age at index date, and year of index date.

© 2024 Hu X et al. *JAMA Network Open.*

eTable 5. Factors Associated With the Likelihood of SUD-Related Visits Among Parents Caring for Children With Cancer

Characteristics	Among Mothers (n=3,887)[a,b]				Among Fathers (n=3,803)[a,c]			
	N	Unadjusted Percent (Row %)	Adjusted Percentage Point Difference (95% CI)	*P*	N	Unadjusted Percent (Row %)	Adjusted Percentage Point Difference (95% CI)	*P*
Parent age group								
25-34 years	597	2.2	Ref		370	1.6	Ref	
35-44 years	1574	1.5	-2.0 (-4.4 to 0.5)	.12	1361	2.1	0.4 (-1.0 to 1.9)	.55
45-54 years	1432	1.2	-2.7 (-5.2 to -0.2)	.03	1580	1.6	0.03 (-1.4 to 1.5)	.97
55-64 years	284	2.3	-2.7 (-5.4 to -0.1)	.04	492	0.8	-1.0 (-2.5 to 0.4)	.15
Parent Charlson Comorbidity Index	---	---	0.5 (0.2 to 0.7)	<.001	---	---	0.1 (-0.4 to 0.6)	.70
Rurality of residence								
Rural	459	1.6	Ref		457	2.6	Ref	
Urban	3428	1.5	0.3 (-0.7 to 1.3)	.53	3346	1.6	-0.7 (-2.0 to 0.5)	.24
US geographic region								
Northeast	888	1.6	Ref		911	1.5	Ref	
Midwest or North Central	914	1.8	0.2 (-0.9 to 1.4)	.71	923	2.4	1.0 (-0.3 to 2.2)	.12
South	1467	1.6	-0.1 (-1.0 to 0.9)	.91	1357	1.8	0.3 (-0.7 to 1.2)	.59
West	618	0.9	-0.5 (-1.6 to 0.6)	.39	612	0.7	-0.6 (-1.7 to 0.4)	.26
Health plan type								
High deductible	522	1.1	Ref		494	1.0	Ref	
HMO	412	1.5	1.0 (-0.7 to 2.7)	.25	404	1.5	0.5 (-1.1 to 2.2)	.51
PPO	2008	1.9	0.7 (-0.3 to 1.8)	.15	1957	1.9	0.6 (-0.6 to 1.7)	.32
Other	945	0.9	-0.05 (-1.1 to 1.0)	.93	948	1.7	0.7 (-0.6 to 2.0)	.28
Number of children in the household								
1	542	2.3	Ref		473	2.5	Ref	
2	1725	1.5	-0.6 (-2.1 to 0.9)	.43	1703	1.5	-0.9 (-2.5 to 0.6)	.24
3 or more	1620	1.3	-0.8 (-2.3 to 0.7)	.31	1627	1.6	-1.1 (-2.6 to 0.5)	.17
Child's sex								
Male	2264	1.6	Ref		2208	1.5	Ref	
Female	1623	1.4	0.001 (-0.7 to 0.8)	.99	1595	1.9	0.3 (-0.5 to 1.1)	.39
Child's age at index cancer diagnosis								
0-4 years	700	1.5	Ref		672	1.5	Ref	
5-14 years	1331	1.1	0.2 (-0.7 to 1.1)	.69	1323	2.0	0.5 (-0.6 to 1.6)	.35

© 2024 Hu X et al. *JAMA Network Open.*

15-21 years	1856	1.8	1.5 (0.2 to 2.8)	.03	1808	1.5	0.2 (-1.0 to 1.4)	.76
Child's cancer type								
Hematologic cancer	1891	1.2	Ref		1793	1.7	Ref	
Bone or connective tissue cancer	512	1.5	0.02 (-1.1 to 1.1)	.97	516	1.4	-0.4 (-1.5 to 0.7)	.48
CNS cancer	888	2.1	1.0 (-0.3 to 2.2)	.14	911	1.6	-0.3 (-1.3 to 0.8)	.60
Gonadal cancer	401	2.1	0.1 (-1.0 to 1.2)	.88	405	1.7	0.6 (-1.2 to 2.5)	.50
Multiple types of cancers	195	1.4	0.1 (-1.7 to 1.9)	.91	178	2.2	0.5 (-1.8 to 2.8)	.68
Child's cancer treatment								
Surgery only	831	1.7	Ref		868	1.6	Ref	
Any HSCT	211	2.1	1.6 (-1.1 to 4.4)	.25	--[c]	--[c]	--[c]	--[c]
Any radiation (no HSCT)	1077	1.6	-0.2 (-1.3 to 0.8)	.65	1092	1.9	0.3 (-0.8 to 1.4)	.58
Any chemotherapy (no HSCT or radiation)	1768	1.3	-0.2 (-1.3 to 0.9)	.69	1843	1.6	0.1 (-1.0 to 1.2)	.89

Notes: The authors' analysis of the Merative™ MarketScan® Commercial Database.
Abbreviations: SUD – substance use and related disorder. CI – confidence interval. Ref. – reference. HMO – health maintenance organization. PPO – preferred provider organization. CNS – central nervous system. HSCT – hematopoietic stem cell transplantation.
[a] Multiple logistic regressions also controlled for parent mental health history and year of index date.
[b] 323 mothers whose child's year of index date was 2010 were dropped from the regression model because of perfect prediction of the outcome (i.e., no SUD-related visit among these 323 mothers).
[c] 213 fathers whose child received HSCT were dropped from the regression model because of perfect prediction of the outcome (i.e., no SUD-related visit among these 213 fathers).

© 2024 Hu X et al. *JAMA Network Open.*

eReferences

1. Cohrs AC, Leslie DL. Depression in Parents of Children Diagnosed with Autism Spectrum Disorder: A Claims-Based Analysis. *J Autism Dev Disord*. 2017;47(5):1416-1422. doi:10.1007/s10803-017-3063-y

2. Ji X, Hu X, Brock KE, Mertens AC, Cummings JR, Effinger KE. Early Post-Therapy Opioid Prescription, Potential Misuse, and Substance Use Disorder among Pediatric Cancer Survivors. *JNCI: Journal of the National Cancer Institute*. Published online 2022:djac049. doi:10.1093/jnci/djac049

3. Hu X, Brock KE, Effinger KE, et al. Changes in Opioid Prescriptions and Potential Misuse and Substance Use Disorders Among Childhood Cancer Survivors Following the 2016 Opioid Prescribing Guideline. *JAMA Oncol*. 2022;8(11):1658-1662. doi:10.1001/jamaoncol.2022.3744

4. *Clinical Classifications Software (CCS) for ICD-9-CM*. Accessed May 24, 2022. https://hcup-us.ahrq.gov/toolssoftware/ccs/ccs.jsp#overview

© 2024 Hu X et al. *JAMA Network Open.*
