# Supplementary material for: Mental Health Care Utilization Among Parents of Children With Cancer
Source: JAMA Netw Open. 2024 Apr 2;7(4):e244531. doi: 10.1001/jamanetworkopen.2024.4531 (PMC10988353; doi:10.1001/jamanetworkopen.2024.4531)
Supplement: Supplement 2. — Data Sharing Statement [file jamanetwopen-e244531-s002.pdf]

Data Sharing Statement

Hu. Mental Health Care Utilization Among Parents of Children With Cancer. *JAMA Netw Open*. Published April 02, 2024. doi:10.1001/jamanetworkopen.2024.4531

Data
Data available: No

Additional Information
Explanation for why data not available: Data for this analysis was made available to the authors through a third-party license from Merative, a commercial data provider in the United States. As such, the authors cannot make these data publicly available due to data use agreement. Other researchers can access the data by purchasing a license through Merative. The inclusion criteria specified in the Methods section would allow other researchers to identify the same cohort of patients used for this analysis. Interested individuals may visit https://marketscan.truvenhealth.com/marketscanportal/ for more information on accessing Merative MarketScan databases.
